# Association of Red Blood Cell Distribution Width With Mortality Risk in Hospitalized Adults With SARS-CoV-2 Infection

**DOI:** 10.1001/jamanetworkopen.2020.22058

**Published:** 2020-09-23

**Authors:** Brody H. Foy, Jonathan C. T. Carlson, Erik Reinertsen, Raimon Padros I. Valls, Roger Pallares Lopez, Eric Palanques-Tost, Christopher Mow, M. Brandon Westover, Aaron D. Aguirre, John M. Higgins

**Affiliations:** 1 Center for Systems Biology, Massachusetts General Hospital, Harvard Medical School, Boston; 2 Department of Pathology, Massachusetts General Hospital, Harvard Medical School, Boston; 3 Department of Systems Biology, Harvard Medical School, Boston, Massachusetts; 4 Cancer Center, Massachusetts General Hospital and Harvard Medical School, Boston; 5 Research Laboratory for Electronics, Massachusetts Institute of Technology, Cambridge; 6 Cardiology Division, Massachusetts General Hospital, Harvard Medical School, Boston; 7 Partners Healthcare Enterprise Research Information Systems, Boston, Massachusetts; 8 Clinical Data AI Center and Neurology Department, Massachusetts General Hospital, Boston; 9 Division of Pulmonary and Critical Care Medicine, Massachusetts General Hospital, Boston; 10 Neurology Department, Harvard Medical School, Boston Massachusetts; 11 Wellman Center for Photomedicine, Massachusetts General Hospital, Harvard Medical School, Boston

## Abstract

**Importance:**

Coronavirus disease 2019 (COVID-19) is an acute respiratory illness with a high rate of hospitalization and mortality. Biomarkers are urgently needed for patient risk stratification. Red blood cell distribution width (RDW), a component of complete blood counts that reflects cellular volume variation, has been shown to be associated with elevated risk for morbidity and mortality in a wide range of diseases.

**Objective:**

To investigate whether an association between mortality risk and elevated RDW at hospital admission and during hospitalization exists in patients with COVID-19.

**Design, Setting, and Participants:**

This cohort study included adults diagnosed with SARS-CoV-2 infection and admitted to 1 of 4 hospitals in the Boston, Massachusetts area (Massachusetts General Hospital, Brigham and Women’s Hospital, North Shore Medical Center, and Newton-Wellesley Hospital) between March 4, 2020, and April 28, 2020.

**Main Outcomes and Measures:**

The main outcome was patient survival during hospitalization. Measures included RDW at admission and during hospitalization, with an elevated RDW defined as greater than 14.5%. Relative risk (RR) of mortality was estimated by dividing the mortality of those with an elevated RDW by the mortality of those without an elevated RDW. Mortality hazard ratios (HRs) and 95% CIs were estimated using a Cox proportional hazards model.

**Results:**

A total of 1641 patients were included in the study (mean [SD] age, 62[18] years; 886 men [54%]; 740 White individuals [45%] and 497 Hispanic individuals [30%]; 276 nonsurvivors [17%]). Elevated RDW (>14.5%) was associated with an increased mortality risk in patients of all ages. The RR for the entire cohort was 2.73, with a mortality rate of 11% in patients with normal RDW (1173) and 31% in those with an elevated RDW (468). The RR in patients younger than 50 years was 5.25 (normal RDW, 1% [n = 341]; elevated RDW, 8% [n = 65]); 2.90 in the 50- to 59-year age group (normal RDW, 8% [n = 256]; elevated RDW, 24% [n = 63]); 3.96 in the 60- to 69-year age group (normal RDW, 8% [n = 226]; elevated RDW, 30% [104]); 1.45 in the 70- to 79-year age group (normal RDW, 23% [n = 182]; elevated RDW, 33% [n = 113]); and 1.59 in those ≥80 years (normal RDW, 29% [n = 168]; elevated RDW, 46% [n = 123]). RDW was associated with mortality risk in Cox proportional hazards models adjusted for age, D-dimer (dimerized plasmin fragment D) level, absolute lymphocyte count, and common comorbidities such as diabetes and hypertension (hazard ratio of 1.09 per 0.5% RDW increase and 2.01 for an RDW >14.5% vs ≤14.5%; *P* < .001). Patients whose RDW increased during hospitalization had higher mortality compared with those whose RDW did not change; for those with normal RDW, mortality increased from 6% to 24%, and for those with an elevated RDW at admission, mortality increased from 22% to 40%.

**Conclusions and Relevance:**

Elevated RDW at the time of hospital admission and an increase in RDW during hospitalization were associated with increased mortality risk for patients with COVID-19 who received treatment at 4 hospitals in a large academic medical center network.

## Key Points

**Question:**

In patients with SARS-CoV-2 infection, is there an association between mortality risk and red blood cell distribution width (RDW), a routine complete blood count component, at the time of admission and during hospitalization?

**Findings:**

In this cohort study of 1641 adult patients with SARS-CoV-2 infection who were hospitalized, elevated RDW at admission and increasing RDW during hospitalization were associated with statistically significant increases in mortality risk. The association between the RDW at admission and mortality risk was independent of D-dimer (dimerized plasmin fragment D) level, absolute lymphocyte count, demographic factors, and common comorbidities.

**Meaning:**

The findings suggest that an elevated RDW measured at admission and increasing RDW during hospitalization were associated with significantly higher mortality risk for patients with SARS-CoV-2 infection; RDW may be helpful for patient risk stratification.

## Teaser

This cohort study assesses the potential use of red blood cell distribution width for risk stratification of patients with coronavirus disease 2019.

## Introduction

Coronavirus disease 2019 (COVID-19) is an acute respiratory illness caused by infection with severe acute respiratory syndrome coronavirus 2 (SARS-CoV-2). COVID-19 has a high rate of hospitalization, critical care requirement, and mortality.^1,2^ Identifying patients at highest risk for severe disease is important to faciliate early, aggressive intervention and to manage local hospital resources to mitigate the critical care crises that have impacted some hospital systems. In general, COVID-19 is associated with lymphopenia, occasional thrombocytopenia, and overall leukopenia at hospital admission.^3^ The clinical course for patients who are hospitalized varies dramatically, with early evidence showing that ICU admission and mortality risk are associated with an elevated D-dimer (dimerized plasmin fragment D) level and a decreasing lymphocyte count.^1,4^ Additional routine biomarkers for patient risk stratification are urgently needed.

The red blood cell distribution width (RDW) is a standard component of a routine complete blood count test. RDW quantifies the variation of individual red blood cell (RBC) volumes, which vary from one cell to the next and for the same cell as it circulates during its approximately 115-day lifespan.^5,6,7^ Elevated RDW is associated with an increased risk for all-cause mortality; mortality from heart disease, pulmonary disease, sepsis, influenza, and cancer; complications associated with heart failure, severity of coronary artery disease and viral hepatitis, advanced stage and grade for many cancers; and the development of diabetes, chronic obstructive pulmonary disease, stroke, anemia, and many other conditions.^8,9,10,11,12,13,14,15,16,17,18^ RDW appears to be a nonspecific marker of illness that has the potential to provide general quantitative risk stratification that may be particularly useful for a new and unknown disease.

RDW is the coefficient of variation in RBC volume, or the SD divided by the mean. An increase in RDW must therefore correspond to a decrease in mean RBC volume (MCV), an increase in RBC volume variance, or both. Previous studies^6,8,17,19^ have found evidence in some specific conditions that RDW elevation is caused by delayed clearance of older RBCs. Because RBCs characteristically decrease in cellular volume across their lifespan, persistence of these older, smaller cells thus increases volume variance, and this clearance delay coincides with and offsets a net decrease in RBC production.^6,8,17^ These reports suggest the possibility that an elevated RDW in some circumstances may reflect a clinical state in which RBC production and turnover have slowed in the setting of increased production and turnover of leukocytes or platelets such as would occur in inflammation. Although a definitive mechanism for RDW elevation has not yet been established, there is evidence that RDW can provide robust risk-stratification among patients diagnosed with the same acute illness. In this study, our aim was to investigate whether an association exists between mortality risk and elevated RDW measured at hospital admission and during hospitalization in patients with COVID-19.

## Methods

### Patients and Study Design

This study was performed in accordance with the Strengthening the Reporting of Observational Studies in Epidemiology (STROBE) reporting guideline. All patient data was gathered using the Partners Healthcare Research Patient Data Registry and Electronic Data Warehouse under a research protocol that was approved for a waiver of patient informed consent by the Partners Healthcare Institutional Review Board because the study involved material collected for nonresearch purposes and involved minimal risk. Clinical data were retrospectively analyzed for all patients who tested positive for SARS-CoV-2 infection between March 4, 2020, and April 28, 2020, at 1 of 4 Partners Healthcare Network hospitals: Massachusetts General Hospital (MGH), Brigham and Women’s Hospital (BWH), North Shore Medical Center (NSMC), and Newton-Wellesley Hospital (NWH) (6376 patients).

Patients were excluded from the study if they were younger than 18 years, or if they did not have an inpatient hospital stay at 1 of the 4 hospitals within 1 month of the initial positive diagnosis (eFigure 1 in the Supplement). A total of 1641 patients (893 from MGH, 446 from BWH, 180 from NSMC, and 122 from NWH) were included in the analysis. Patients with multiple separate inpatient visits related to COVID-19 were treated as having been admitted during the first visit and discharged at the final visit. For analysis purposes, patients who had visits spanning multiple medical centers were classified as being in the cohort associated with the first medical center where they were admitted after a COVID-19 diagnosis. The final patient discharge occurred on June 26, 2020, with no COVID–19-related readmissions occurring by July 25, 2020 (the data collection end point).

For all inpatients, RDW, absolute lymphocyte count, and D-dimer level were collected approximately daily along with other clinical laboratory values, as part of standard clinical care. Complete blood counts, including RDW and lymphocyte count were performed on an XN-9000 Automated Hematology System (Sysmex Corporation). D-dimer level was measured using a Vidas 3 immunoanalyzer (bioMérieux). SARS-CoV-2 infection was diagnosed using multiple instruments and assays including the bioMérieux BioFire Respiratory 2.1 panel, Roche Cobas 6800 system, and Cepheid GeneXpert molecular diagnostic system.

Race and ethnicity were self-reported by patients and were obtained from medical records. Race was categorized as one of the following categories: Black/African American; White; other or unknown (encompassing Asian, Pacific Islander or Hawaiian, Native American Indian or Alaskan native, Hispanic/Latino (some patients self-reported Hispanic/Latino race), other, and declined to respond or unknown). Ethnicity was categorized as one of the following categories: Hispanic; non-Hispanic; unknown (declined to respond or unknown). These categorizations were chosen and included in the analysis because previous reports have suggested potential differences in infection risk and disease severity among these groups.^20,21,22,23^ Comorbidities were analyzed by identifying *International Statistical Classification of Diseases and Related Health Problems, Tenth Revision* codes associated with the diagnostic history of each patient. Mortality was determined by reviewing discharge summaries, with an assumption of no COVID–19-related deaths for patients who were discharged alive. The implications of this assumption are explored in the Supplement.

Results are presented using pooled data from MGH, BWH, NSMC, and NWH. Similar results were found when the BWH and MGH cohorts were each analyzed separately (eFigures 2 and 3 and eTable 1 in the Supplement); NSMC and NWH cohorts were not analyzed separately because of their small size.

### Statistical Analysis

The Kaplan-Meier method was used to analyze survival in inpatients who were stratified by RDW at admission. To account for age as a potential confounder and the potential for effect modification, patients were categorized into 5 age groups: <50 years, 50 to 60 years, 60 to 70 years, 70 to 80 years, and ≥80 years. There were 206 patients younger than 40 years, of which 2 died. An elevated RDW was defined as greater than 14.5%, the current upper limit of the healthy adult reference interval at both MGH and BWH. Patients who were discharged alive were censored on June 26, 2020 (the date of last discharge across the cohort). The implications of censoring at discharge are presented in eFigure 4 in the Supplement.

Mortality hazard ratios (HRs) were calculated using a Cox proportional hazards model. Models were fit with univariate inputs and multivariate inputs, using RDW, age, race, and ethnicity, and 2 clinical COVID-19 risk factors: absolute lymphocyte count, and D-dimer level.^4^ Owing to known demographic risk profiles, race and ethnicity were encoded as binary variables to compare higher risk groups with lower risk groups.^20^ Race was coded as 1 for Black/African American, and 0 for all other groups. Ethnicity was coded as 1 for Hispanic, and 0 for non-Hispanic/unknown. Models were fit with variables catgorized as either continuous or binary using a risk threshold. Risk thresholds were defined as age older than 70 years, RDW greater than 14.5%, absolute lymphocyte count <0.8 × 10^9^/L, and D-dimer level greater than 1500 ng/mL (to convert to nanomoles per liter, multiply by 0.005476). Thresholds for age, absolute lymphocyte count, and D-dimer level were chosen based on previous COVID-19 studies.^4^ Using these thresholds, each high-risk cohort was of a similar size: age older than 70 years (30% of patients), RDW greater than 14.5% (34% of patients), lymphocyte count less than 0.8 × 10^9^/L (26% of patients), D-dimer level higher than 1500 ng/L (26% of patients). For continuous models, HRs were normalized based on clinically meaningful changes in the measurements: 10 years for age, 0.5% for RDW, 0.1 x10^9^/L for absolute lymphocyte count, and 100 mg/L for D-dimer level. For ease of comparison, HRs for absolute lymphocyte count were inverted to represent the increased risk for a decrease in value. All other HRs were relative to increases. Multivariate proportional hazards models were also fit using patient comorbidities, with results presented in the eTable 3 in the Supplement. To account for potential effect modification, models were fit separately for each age category (eTable 2 in the Supplement). For completeness, models were also fit incorporating other blood count measures (eTable 4 in the Supplement).

Changes in RDW during the hospital stay were evaluated by taking the percentage point difference between the first and last available RDW measurement. All patients had at least 2 distinct RDW measurements during their stay. RDW trajectories were plotted for patients and were stratified by normal and abnormal RDW as well as survival status on discharge. Mean RDW trajectories were calculated by linearly interpolating patient RDW values and calculating the mean value for all patients in a cohort at the interpolated time after admission (curves were calculated using a temporal spacing of 1 hour). Mean RDW trajectories were calculated across the first week of admission, and cohorts were limited to patients who had a hospital stay of at least 7 days. The implications of this exclusion are explored in eFigure 5 and 6 in the Supplement.

Statistical differences between cohorts were analyzed using a χ^2^ proportion comparison test for incidence rates (%), a 2-sample, 2-sided *t* test for means, and a 2-sided Wilcoxon rank sum test for medians. Differences in HRs and RRs were analyzed using a Mantel-Haenszel test.^24^ All statistical analysis was performed using MATLAB 2019b (MathWorks). *P* < .05 was considered to be statistically significant.

## Results

### Baseline Characteristics of Patients

We retrospectively investigated RDW measured at the time of admission for a diagnosis of SARS-CoV-2 infection in 1641 patients admitted to 1 of 4 hospitals in the Boston, Massachusetts area between March 4, 2020, and April 28, 2020. The mean (SD) age of the patients was 62 (18) years, and 886 were men (54%). Of 1641 patients, 740 were White individuals (45%) and 497 were Hispanic individuals (30%). Of 1641 patients, 276 died (17%). Other baseline characteristics of patients, stratified by survival status, are shown in Table 1.

**Table 1.** Patient Characteristics Stratified by Mortality at Discharge

Demographic Characteristics	Mean (SD)		*P* value^a^
	Survivors	Nonsurvivors	
No.	1365	276	
Age, y	59.6 (17.6)	74.6 (13.4)	<.001
Male, No. (%)	723 (53)	163 (59)	.07
BMI	30.8 (6.8)	30.2 (7.2)	.21
Race, No. (%)			
White/Caucasian	585 (43)	155 (56)	<.001
Black/African American	223 (16)	58 (21)	.04
All other/unknown	41	23	<.001
Ethnicity, No. (%)			
Hispanic	448 (33)	49 (18)	<.001
Non-Hispanic/unknown	67	82	<.001
RDW % stratified by age group			
<50	13.4 (1.9)	15.8 (3.8)	<.001
50-59 y	13.6 (2.1)	14.8 (2.6)	.002
60-69 y	13.8 (1.6)	15.5 (2.2)	<.001
70-79 y	14.1 (1.8)	14.7 (2.0)	.03
≥80	14.2 (1.6)	15.0 (1.8)	<.001
Entire cohort	13.8 (1.8)	15.0 (2.2)	<.001
Other laboratory tests			
Absolute lymphocyte count, N × 10^9^/L	1.24 (2.98)	1.05 (1.61)	.33
Dimerized plasmin fragment D, median (IQR), ng/L	845 (498-1551)^b^	1282 (635-2123)^b^	<.001
Hematocrit, %	39.2 (5.7)	37.7 (7.0)	<.001
Hemoglobin, g/dL	13.0 (2.0)	12.2 (2.4)	<.001
Mean corpuscular hemoglobin, pg/cell	29.0 (2.5)	29.3 (2.7)	.04
Mean corpuscular hemoglobin concentration, g/dL	33.1 (1.4)	32.4 (1.6)	<.001
Platelet count, 10^3^/μL	216.8 (92.4)	185.4 (90.4)	<.001
Red blood cell count, 10^6^/μL	4.5 (0.7)	4.2 (0.9)	<.001
White blood cell count, 10^3^/μL	7.4 (6.3)	8.1 (4.3)	.05
Other outcomes			
Length of hospital stay, d	16.7 (15.5)	11.8 (11)	<.001
Comorbidities, %			
Any	37	54	<.001
COPD	4	13	<.001
Diabetes	17	22	.04
Hypertension	23	36	<.001
Coronary artery disease	8	16	<.001
Chronic kidney disease	8	21	<.001

Abbreviations: BMI, body mass index (calculated as weight in kilograms divided by height in meters squared); COPD, chronic obstructive pulmonary disease; IQR, interquartile range; RDW, red blood cell distribution width.

SI conversion: To convert dimerized plasmin fragment D level to nmol/L, multiply by 0.005476; to convert platelet count to ×10^9^/L, multiply by 1.0; to convert red blood cell count to ×10^12^/L, multiply by 1.0; to convert white blood cell count to ×10^9^/L, multiply by 0.001.

^a^ Statistical significance was calculated using a 2-sided *t* test for means, a χ^2^ test for percentages, and 2-sided Wilcoxon rank sum test for dimerized plasmin fragment D.

^b^ Dimerized plasmin fragment D is presented as median IQR because of its long upper tail.

### Elevated RDW at Admission and Mortality Risk

**Figure 1.**
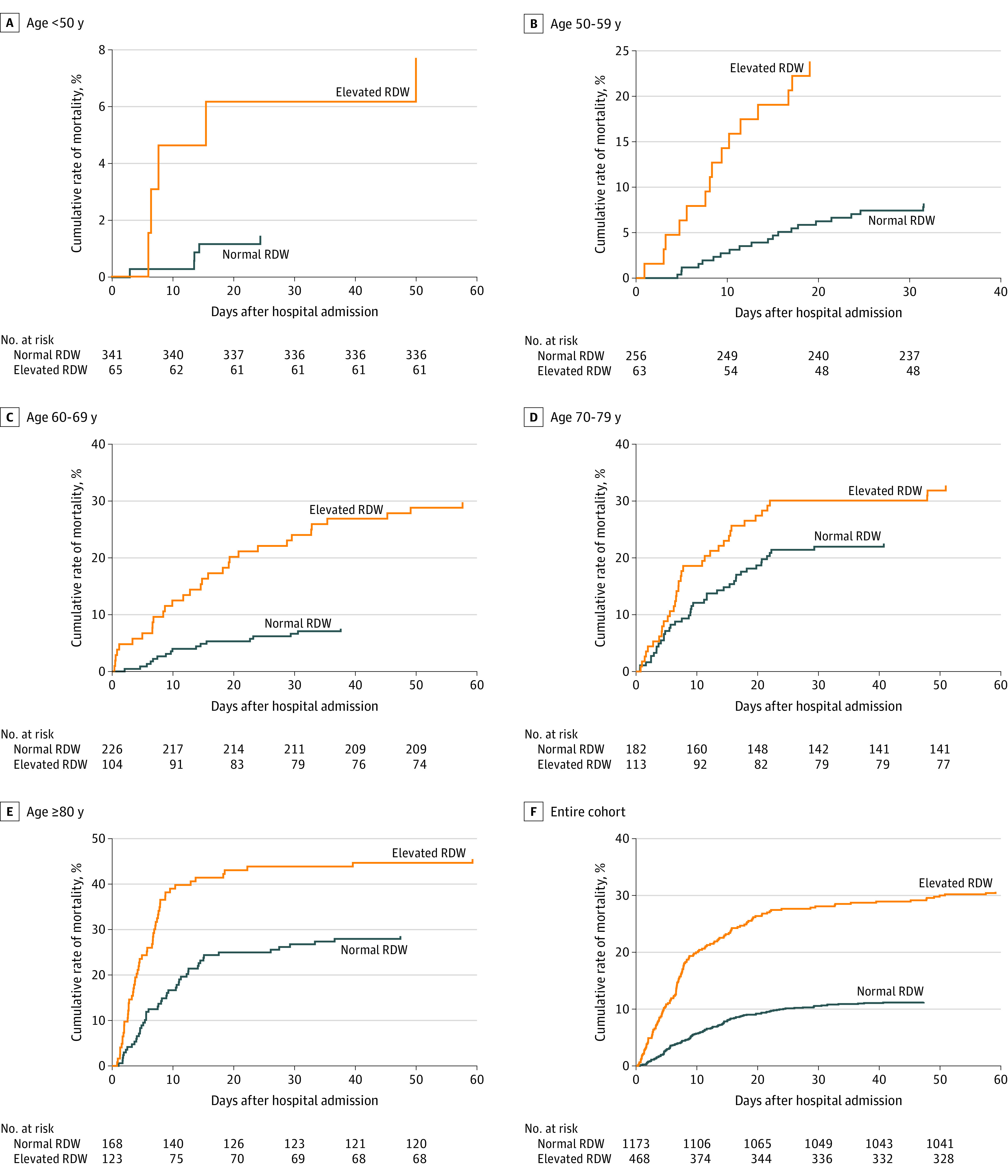
Elevated Red Blood Cell Distribution Width (RDW) at Hospital Admission and Mortality Among Patients With Coronavirus Disease 2019 Across all age groups, an RDW greater than 14.5% measured at the time of admission was associated with a 31% mortality compared with an 11% mortality for patients whose RDW at admission was ≤14.5%. All increases in mortality are statistically significant except in the 70- to 80-year age group. Table 2 details age-stratified and RDW-stratified mortality rates.

Patients whose RDW was greater than 14.5% at admission for a COVID-19 diagnosis had a mortality risk of 31%, whereas those with an RDW of 14.5% or less had a mortality risk of 11%. The RR of mortality for those with an elevated RDW was 2.73 (95% CI, 2.52-2.94). Age has previously been shown to be a risk factor for COVID-19 mortality.^4^ In patient groups stratified by age, elevated RDW remained associated with increased relative risk of mortality for patients younger than 50 years, 50 to 59 years, 60 to 69 years, and 80 years or older (Figure 1 and Table 2). For patients in the 70- to 79- year age group, the RR was elevated (1.45) but was not statistically significant. Relative risk was particularly elevated within 48 hours of admission, with patients with a normal RDW of 14.5% or less having a mortality of 0.8% (9 of 1175 patients) within 48 hours of admission, whereas the mortality rate for those with an RDW greater than 14.5% was 4.9% (23 of 470 patients), a risk ratio of 6.12. Risk ratios for different age groups were significantly different compared with each other, suggesting an effect modification, with an elevated RDW having a larger effect on mortality for younger patients (<70 years) than it had for older patients.

**Table 2.** Mortality Rates Stratified by Age and RDW Elevation at Admission

Age group, y	Normal RDW		Elevated RDW^a^		*P* value	Risk ratio (95% CI)^b^
	No.	Mortality, %	No.	Mortality, %		
<50	341	1	65	8	.003	5.25 (4.04-6.46)
50-59	256	8	63	24	<.001	2.90 (2.30-3.51)
60-69	226	8	104	30	<.001	3.96 (3.42-4.51)
70-79	182	23	113	33	.05	1.45 (1.08-1.83)
≥80	168	29	123	46	.003	1.59 (1.29-1.90)
Entire cohort	1173	11	468	31	<.001	2.73 (2.52-2.94)

Abbreviation: RDW, red blood cell distribution width.

^a^ Elevated RDW was considered to be greater than 14.5%.

^b^ Risk ratios were statistically significantly different (*P* < .001) from each other (on the basis of a Mantel-Haenszel test), suggesting that patients younger than 70 years had higher risk ratios.

### Association of RDW with Mortality Risk After Adjustment for Age, Race, Ethnicity, D-dimer Level, and Lymphopenia

**Figure 2.**
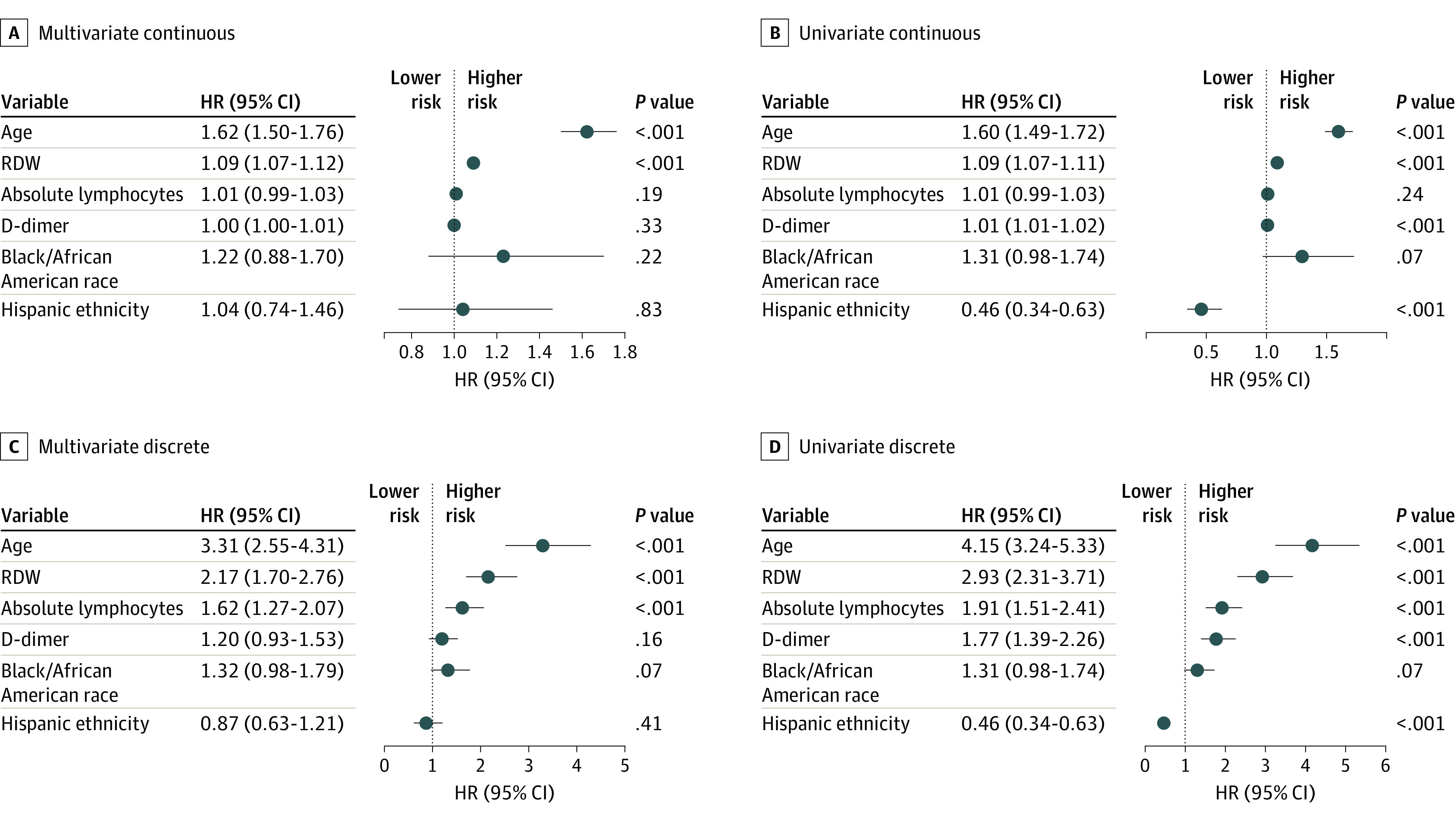
Cox Proportional Hazards Modeling of Mortality Risk Models of mortality adjusted for age, race, ethnicity, red blood cell distribution width (RDW), absolute lymphocyte count, and D-dimer (dimerized plasmin fragment D) level are given for the multivariate (A) and univariate (B) analyses. Variables were coded as either continuous (A and B) or discrete (C and D) using the following thresholds: age older than 70 years, RDW >14.5%, lymphocyte count <0.8 × 10^9^/L, and D-dimer level greater than 1500 ng/L, which provided similar proportions of abnormality in the cohort (33%, 29%, 27%, and 28%, respectively, for age, RDW, lymphocyte count, and D-dimer level). Race was coded as 1 for Black/African American, and 0 for all other groups. Ethnicity was coded as 1 for Hispanic, and 0 for non-Hispanic/unknown. For continuous models, changes in variables were normalized as follows: age increase of 10 years, RDW increase of 0.5%, D-dimer level increase of 100 ng/L, and a lymphocyte count decrease of 0.1 103 ×10^9^/L.

Previous studies have found an elevated D-dimer level and low absolute lymphocyte count to be associated with an increased mortality risk.^4^ Cox proportional hazards regression modeling was used to investigate whether RDW provided independent risk information beyond these markers, both when considered as a binary marker relative to the 14.5% reference interval boundary and when considered as a continuous marker. Figure 2 shows that RDW greater than 14.5% was associated with a statistically significant increased risk of mortality for all models considered, including those adjusted for age, race, ethnicity, absolute lymphocyte count, and D-dimer level as continuous and binary variables. Age and RDW were the only variables with statistically significant risk ratios for both the continuous and discrete multivariate models. Black/African American race appeared to be associated with elevated risk of mortality in the multivariate discrete model, but the results were not statistically significant. Hispanic ethnicity was associated with a lower risk of mortality in the univariate model, but not in the multivariate model, likely reflecting the lower mean (SD) age of Hispanic patients (54 [16.2] years) compared with the mean (SD) age of other patients (66 [17.1] years). The statistical significance of the association between mortality and RDW persisted when separate Cox proportional hazards models were fit for each age group (eTable 2 in the Supplement).

We performed additional Cox proportional hazards modeling (eTable 3 in the Supplement), incorporating 5 major comorbidities (chronic obstructive pulmonary disease (COPD), coronary artery disease, chronic kidney disease (CKD), diabetes, and hypertension). When jointly modeled, the HR associated with an RDW greater than 14.5% remained statistically significant and was greater than that for any comorbidity (HR, 2.01 [95% CI, 1.57-2.57]; *P* < .001), and RDW modeled as a continuous variable was statistically significant with a HR of 1.09 per 0.5 percentage point increase in RDW. When accounting for RDW, CKD and COPD were the only comorbidities that retained statistically significant HRs (CKD, 1.66 [95% CI, 1.20-2.29]; *P* = .002; COPD, 1.69 [95% CI, 1.18-2.44]; *P* = .005]. When multivariate models included some other blood count measures (eTable 4 in the Supplement), only RDW and platelet (PLT) count had statistically significant HRs (RDW >14.5%, 2.04 [95% CI, 1.55-2.69]; PLT<150 × 10^3^/μL, 1.76 [95% CI, 1.37-2.25]; *P* < .001).

### Increasing RDW After Hospital Admission and Mortality Risk

**Figure 3.**
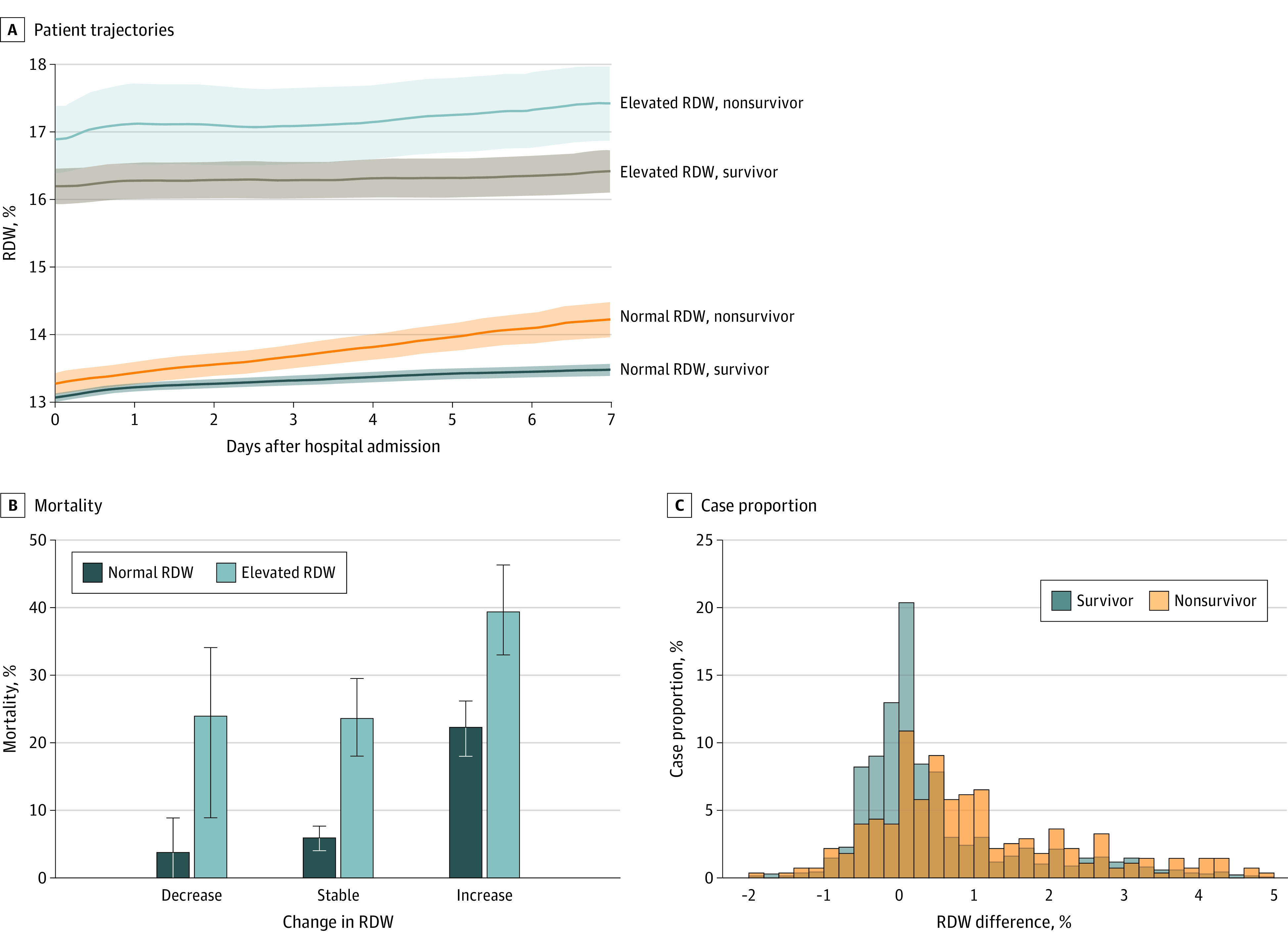
Red Blood Cell Distribution Width (RDW) Increase After Admission and Mortality Risk Among Patients With Coronavirus Disease 2019 A, Stratifying patients based on admission RDW and mortality reveals that, among patients with an RDW of 14.5% or less at admission, those who do not survive have an average RDW increase of 1.5% during their first week of hospitalization, a significantly larger RDW increase than in all other groups. Shading represents the 95% CI. B, Among patients with an RDW of 14.5% or less at admission, those with an increase of more than 0.5% in RDW between admission and discharge had a 24% (95% CI, 18%-30%) mortality rate compared to 6% (95% CI, 4%-8%) for those with stable RDW (≥−0.5% and ≤0.5%). Among patients with elevated RDW at admission, a further increase in RDW during admission was associated with a mortality rate of 40% (95% CI, 33%-47%), and a stable elevated RDW was associated with a mortality rate of 22% (95% CI, 18%-26%). C, A histogram of RDW change in survivors and nonsurvivors of coronavirus disease 2019 shows that nonsurvivors were more likely than survivors to experience an RDW increase during hospitalization. Change in RDW is reported in percentage points. For instance, a change in RDW from 14.0% to 15.0% is reported as 1.0%.

We investigated whether changes in RDW after admission were associated with increased mortality risk for those with initially elevated RDW at hospital admission and those with normal RDW. Figure 3 shows that patients with an RDW of 14.5% or less at admission who died had an increasing mean RDW, whereas those with an RDW of 14.5% or less who were alive at discharge had a stable RDW. For all patients, an increasing RDW during hospitalization was associated with increased mortality risk: from 6% (95% CI, 4%-8%) to 24% (95% CI, 18%-30%) for those with a normal RDW at admission and from 22% (95% CI, 18%-26%) to 40% (95% CI, 33%-47%) for those with an elevated RDW at admission. Figure 3 shows that the mean RDW in the elevated group is 16.4 compared with 13.0 in the nonelevated group. In general, a 1.25-fold higher RDW reflects a 1.25-fold smaller MCV, a 1.25-fold larger SD of the RBC volume distribution, or a combination of smaller changes to both. The average MCV differed by a factor of only about 1.01 (89 fL vs 87.9 fL) (eFigures 5 and 6 in the Supplement). Although the true baseline RDW for the elevated RDW group is unknown, these results suggest that the major contributor to elevated RDW is an increase in variance of the RBC volume distribution instead of a decrease in MCV. Figure 3 also shows that those who do not survive have an average RDW increase of 1.5% during their first week of hospitalization, a significantly larger RDW increase than in all other groups. Few patients experienced greater than a 2% increase per week in RDW during their hospitalization, and the large increase in the elevated RDW group raises the possibility of a longer duration of disease for these patients at the time of admission.

## Discussion

In this cohort study, an RDW greater than 14.5% at the time of admission for SARS-CoV-2 infection was associated with an increase in mortality risk (from 11% to 31%) in a cohort of 1641 patients treated at a large academic medical center network. Risk of mortality associated with RDW remained statistically significant after adjustment for patient age, race, ethnicity, D-dimer level, absolute lymphocyte count, other blood count measures, and 5 major comorbidities. Patients whose RDW increased during admission also had an increased mortality risk. RDW is routinely measured and may be helpful for prioritizing patients for early, aggressive intervention and managing local hospital resource use.

Patients with elevated RDW at admission were 6.12 times more likely to die within 48 hours (23 of 470 patients [4.9%]) than patients with a normal RDW (9 of 1175 patients [0.8%]). This rapid decompensation is consistent with hospital presentation after substantial disease progression, but timing of disease onset was not available for this cohort. Elevated RDW resulted in a larger increase in mortality risk in younger patients (<70 years) compared with older patients. This finding may reflect the higher overall mortality rate for older patients, dampening an RDW effect measured in terms of relative risk. A contributing factor may be the lower RDW at admission in the younger age groups (Table 1), which implies that an RDW >14.5% represents a larger change compared with baseline RDW in these younger patients. It is also possible that an RDW greater than 14.5% is a stronger inflammatory marker in younger patients than in older patients, but studies that are specifically designed to test this hypothesis are required. Race and ethnicity were not statistically significantly associated with an increased mortality risk after adjusting for age and RDW, suggesting that neither race nor ethnicity had implications for patient outcomes after admission. Patients who self-reported Black/African American race or Hispanic ethnicity were overrepresented in the admitted hospital cohort (17% Black/African American and 30% Hispanic) compared with 9% and 12% in a Massachusetts population,^25^ consistent with a higher risk of infection in these racial and ethnic groups.^20,23^

Other studies have noted the potential value of using RDW for a differential diagnosis of pneumonia^26,27^ or as a marker of complication rates in SARS-CoV-2 infection,^28,29,30,31^ either directly or as a component of a machine learning framework. Recent small-scale studies have included RDW in multivariate models along with neutrophil-lymphocyte ratio^28^ or hemoglobin^26^ for a differential diagnosis of COVID-19. The results of the present study show that the use of RDW as a univariate marker relative to its predetermined reference interval (≤14.5%) is associated with substantially increased mortality risk. We also found that RDW was associated with the highest risk ratio when considered in multivariate models with some other blood count measures (eTable 4 in the Supplement). The only other blood count measure studied with a statistically significant risk ratio was PLT count, and future investigation is warranted given the evidence of thrombotic complications within COVID-19 patients.^32,33^

The specific mechanism or mechanisms for the RDW alteration associated with COVID-19 remain unclear. RDW is a nonspecific marker of general illness^8,9,10,11,12,13,14,15,16,17^ and is therefore unlikely to be causally associated with COVID-19 disease progression. COVID-19 is associated with altered turnover in all WBC lineages, as noted previously, as well as with altered platelet dynamics in COVID-associated coagulopathy.^32^ The association of elevated RDW with COVID-19 severity could be consistent with previous reports (in non–COVID-19 cohorts), suggesting that RDW can become elevated when RBC production kinetics have slowed in the setting of increased WBC and platelet kinetics.^6,8,17^

It is unknown whether patients admitted with an RDW greater than 14.5% had higher baseline RDW than those who were admitted with an RDW of 14.5% or less before SARS-CoV-2 infection. RDW usually changes slowly because it reflects the volume variance of a cell population that is turning over at a rate typically no larger than a 1% or 2% per day. The large increase in the elevated RDW group (>3% shown in Figure 3A) may suggest a longer duration of disease for these patients at the time of admission, but direct study of the earlier phases of the disease is required to know how quickly RDW may be evolving before hospitalization, and determination of time of initial infection was not possible within this study cohort.

Patients with many different underlying acute and chronic illnesses would be expected to have a higher baseline RDW, and it is possible that the RDW measured at admission is a nonspecific summary marker of the presence of these illnesses that have been shown to be associated with elevated RDW and may be expected to complicate the COVID-19 clinical course. Regardless of the reasons for the differences in RDW at admission, the association of elevated RDW with increased mortality risk appears to persist after admission, as demonstrated by the higher mortality rate for patients in the present cohort whose RDW increased during hospitalization.

### Limitations

This study has limitations. It describes the potential value of RDW for risk stratification of admitted patients with COVID-19, but because the present analysis was limited to a hospitalized cohort, these results may not apply to individuals with COVID-19 who are not hospitalized. Because the time of initial infection was unavailable, these results are not specific to any disease progression time points. The cohort may have been underpowered for evaluation of mortality risk associated with self-reported Black/African American race. In addition, the socioeconomic status^20,21,22,23^ of patients was unavailable, and the potential association of mortality risk with socioeconomic status could not be assessed. The study included few younger patients (206 patients younger than 40 years, 2 deaths), and results should only be considered valid for those older than 40 years. In addition, although results in this study spanned 4 hospitals, they are located in the same geographic region (Boston, Massachusetts). Although other studies support the potential value of RDW for risk stratification in inflammatory scenarios,^8,9,10,11,12,13,14,15,16,17^ this study cohort may not be representative of other US and non-US populations.

## Conclusions

In this cohort study of patients hospitalized for COVID-19, RDW measured at admission and during hospitalization was associated with a statistically significant increase in mortality. RDW is a routine laboratory test that may be useful in risk stratification of hospitalized patients with COVID-19.

## Notes

Supplement. **eFigure 1.** Cohort exclusion diagram **eTable 1.** Age and RDW-stratified mortality rates upon discharge at MGH and BWH **eFigure 2.** Age and RDW-stratified mortality curves at Massachusetts General Hospital **eFigure 3.** Age and RDW-stratified mortality curves at Brigham and Women’s Hospital **eFigure 4.** Age and RDW-stratified mortality curves, censoring patients upon hospital discharge **eTable 2.** Multivariate Cox proportional-hazards analysis stratified by age **eTable 3.** Multivariate Cox proportional-hazards analysis including common comorbidities **eTable 4.** Cox proportional-hazards analysis including blood count measures **eFigure 5.** Mean MCV trajectories for inpatients, stratified by RDW and mortality **eFigure 6.** Mean RDW trajectories for inpatients, stratified by RDW and mortality, including short stay patients
