# Supplementary material for: Association of Red Blood Cell Distribution Width With Mortality Risk in Hospitalized Adults With SARS-CoV-2 Infection
Source: JAMA Netw Open. 2020 Sep 23;3(9):e2022058. doi: 10.1001/jamanetworkopen.2020.22058 (PMC7512057; doi:10.1001/jamanetworkopen.2020.22058)
Supplement: Supplement. — eFigure 1. Cohort exclusion diagram eTable 1. Age and RDW-stratified mortality rates upon discharge at MGH and BWH eFigure 2. Age and RDW-stratified mortality curves at Massachusetts General Hospital eFigure 3. Age and RDW-stratified mortality curves at Brigham and Women’s Hospital eFigure 4. Age and RDW-stratified mortality curves, censoring patients upon hospital discharge eTable 2. Multivariate Cox proportional-hazards analysis stratified by age eTable 3. Multivariate Cox proportional-hazards analysis including common comorbidities eTable 4. Cox proportional-hazards analysis including blood count measures eFigure 5. Mean MCV trajectories for inpatients, stratified by RDW and mortality eFigure 6. Mean RDW trajectories for inpatients, stratified by RDW and mortality, including short stay patients [file jamanetwopen-e2022058-s001.pdf]

Supplementary Online Content

Foy BM, Carlson JCT, Reinertsen E, et al. Association of red blood cell distribution width with mortality risk in hospitalized adults with SARS-CoV-2 infection. *JAMA Netw Open*. 2020;3(9):e2022058. doi:10.1001/jamanetworkopen.2020.22058

eFigure 1. Cohort exclusion diagram

eTable 1. Age and RDW-stratified mortality rates upon discharge at MGH and BWH

eFigure 2. Age and RDW-stratified mortality curves at Massachusetts General Hospital

eFigure 3. Age and RDW-stratified mortality curves at Brigham and Women's Hospital

eFigure 4. Age and RDW-stratified mortality curves, censoring patients upon hospital discharge

eTable 2. Multivariate Cox proportional-hazards analysis stratified by age

eTable 3. Multivariate Cox proportional-hazards analysis including common comorbidities

eTable 4. Cox proportional-hazards analysis including blood count measures

eFigure 5. Mean MCV trajectories for inpatients, stratified by RDW and mortality

eFigure 6. Mean RDW trajectories for inpatients, stratified by RDW and mortality, including short stay patients

This supplementary material has been provided by the authors to give readers additional information about their work.

© 2020 Foy BM et al. *JAMA Network Open.*

eFigure 1 Cohort exclusion diagram

The cohort exclusion diagram associated with the main study is presented in **eFigure 1**. Initially all patients who tested positive for SARS-CoV-2 at a Partners network hospital, between March 4th-April 28th 2020 were identified (N = 6,376). 4,708 patients were excluded for having no inpatient stay at a Partners Network hospital. A further 20 patients were excluded due to their hospital stay starting more than 30 days after COVID-19 diagnosis (meaning the stay was likely not directly caused by the COVID-19 infection), and 4 patients were excluded as they were under 18 years old. These exclusions led to a final cohort of 1641 patients across four medical centers: Massachusetts General Hospital (MGH), Brigham and Women's Hospital (BWH), North Shore Medical Center (NSMC), and Newton-Wellesley Hospital (NWH).

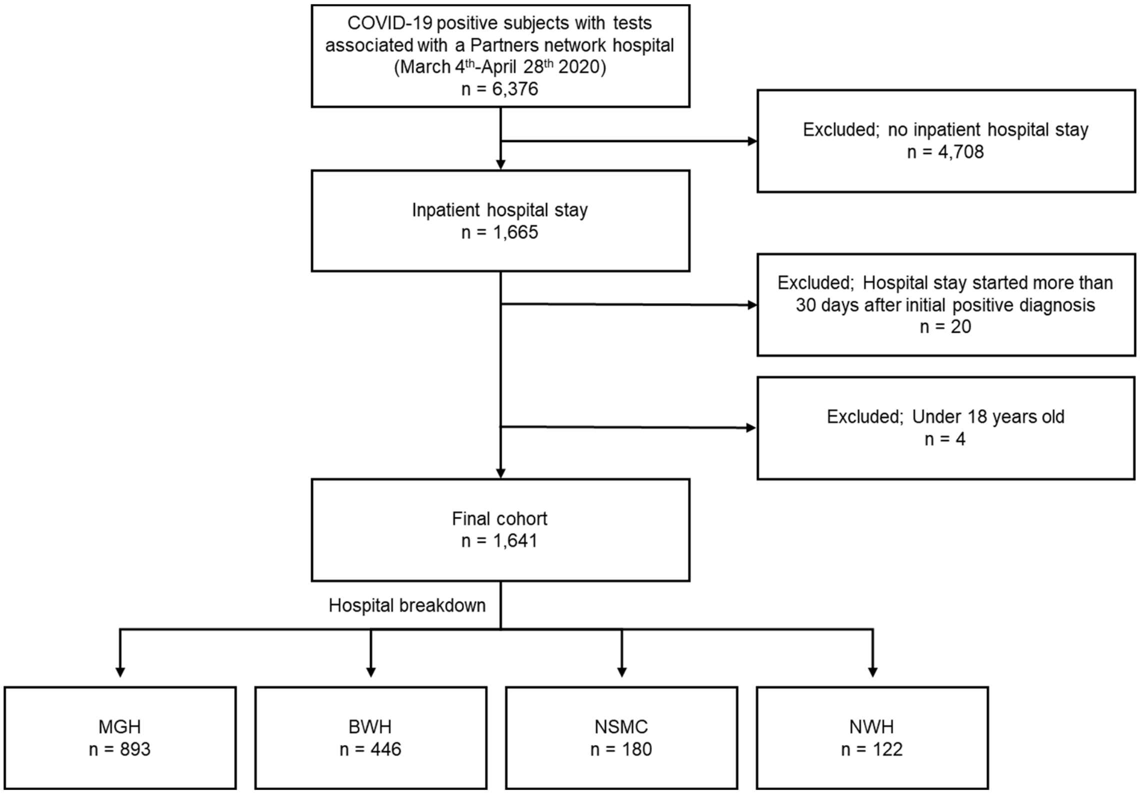

© 2020 Foy BM et al. *JAMA Network Open.*

Survival curves stratified by hospital

Within the main manuscript, results were presented using a patient cohort pooled from four separate medical centers. To illustrate robustness of the findings, here we recreate key results separately for MGH and BWH (NSMC and NWH were omitted due to cohort sizes being too small for statistical power). Within **eFigure 2** and **eFigure 3** we present the Kaplan-Meier survival curves for MGH and BWH respectively. Mortality at discharge and RDW-stratified risk ratios corresponding to **eFigure 2-3** are given in **eTable 1.**

As these results show, results are broadly consistent with the main study findings. Patients with elevated RDW (>14.5%) upon hospital admission had higher mortality rates than those in the normal RDW cohort, for all age groups. However, differences in mortality rates between normal and elevated RDW cohorts were not statistically significant for <50yrs, and 70-80yrs at MGH, and <50yrs, 70-80yrs, and 80+yrs at BWH, consistent with the smaller cohort sizes of between 24-51 patients in the elevated RDW age-stratified groups.

eTable 1. Age and RDW-stratified mortality rates upon discharge at MGH and BWH

Massachusetts General Hospital						
	Normal RDW		Elevated RDW			
Age	N	Mortality	N	Mortality	p-value	Risk Ratio (95% confidence interval)
<50yrs	241	2%	32	3%	0.56	1.88 (0.00-4.04)
50-60yrs	157	3%	27	19%	0.001	5.81 (4.64-6.99)
60-70yrs	127	7%	44	30%	< 0.001	4.17 (3.39-4.95)
70-80yrs	92	23%	49	31%	0.31	1.34 (0.78-1.91)
80+ yrs	73	27%	51	45%	0.04	1.65 (1.17-2.13)
Entire cohort	690	9%	203	28%	< 0.001	3.28 (2.96-3.61)
Brigham and Women's Hospital						
	Normal RDW		Elevated RDW			
Age	N	Mortality	N	Mortality	p-value	Risk Ratio (95% confidence interval)
<50yrs	52	0%	24	4%	0.14	N/A
50-60yrs	61	15%	29	34%	0.03	2.34 (1.55-3.12)
60-70yrs	59	8%	42	31%	0.004	3.65 (2.70-4.60)
70-80yrs	51	27%	39	41%	0.18	1.49 (0.91-2.08)
80+ yrs	49	24%	40	40%	0.12	1.63 (1.01-2.25)
Entire cohort	272	15%	174	32%	< 0.001	2.19 (1.83-2.55)

© 2020 Foy BM et al. *JAMA Network Open.*

eFigure 2. Age and RDW-stratified mortality curves at Massachusetts General Hospital.

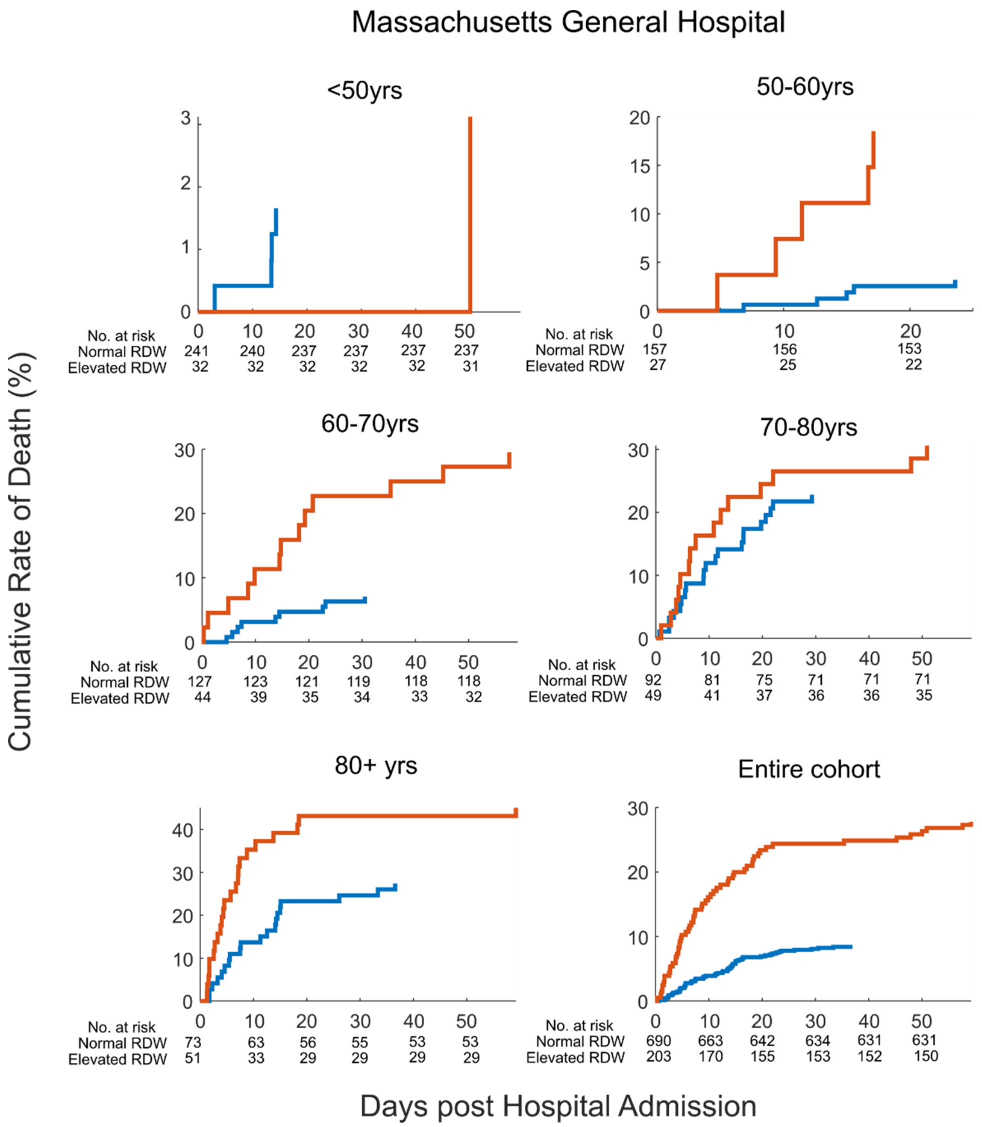

Patient data was censored based on the maximum discharge date in the cohort (June 26th, 2020), reflecting an assumption that patients discharged alive have no mortality due to covid-19 at a later date.

© 2020 Foy BM et al. *JAMA Network Open.*

eFigure 3. Age and RDW-stratified mortality curves at Brigham and Women's Hospital

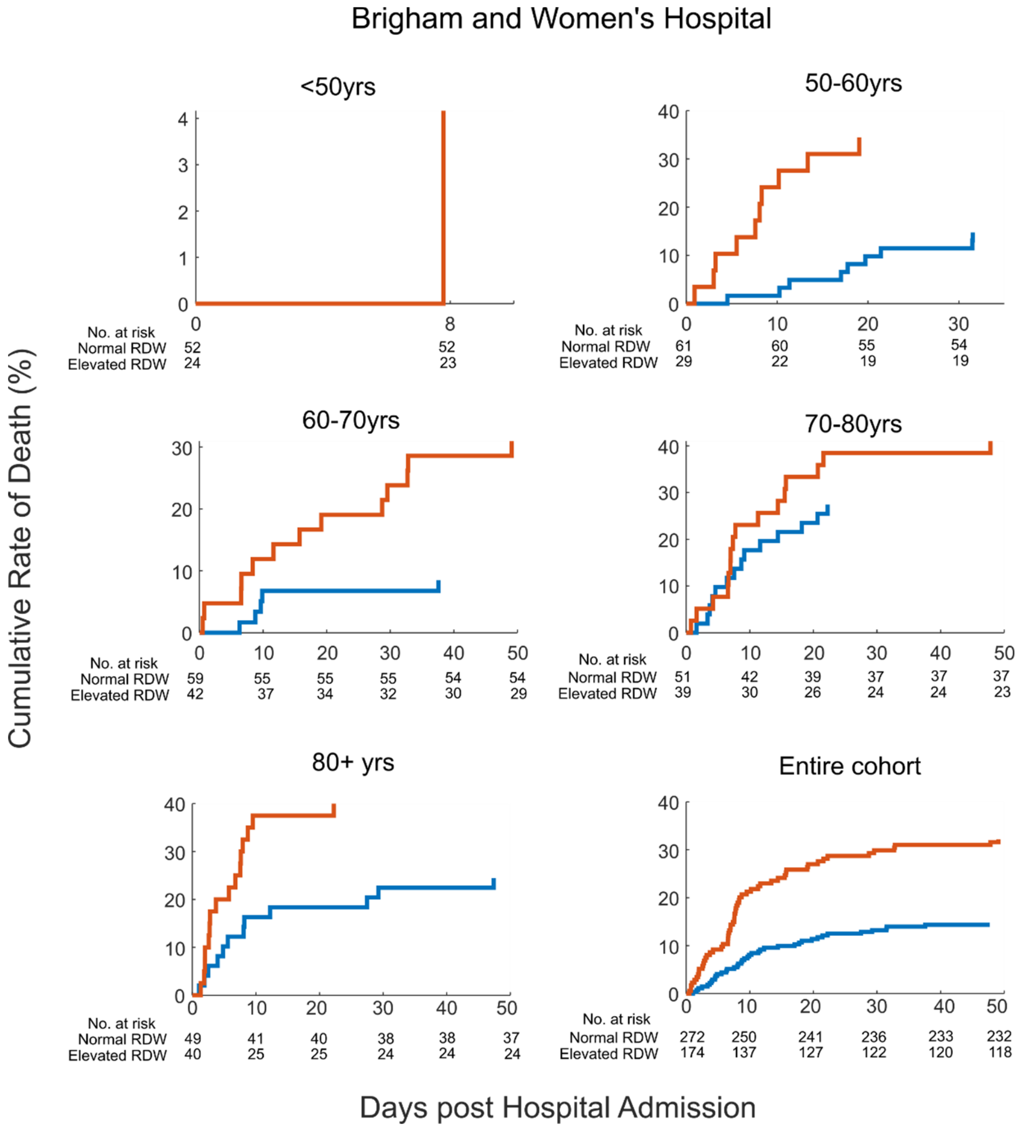

Patient data was censored based on the maximum discharge date in the cohort (June 26th, 2020), reflecting an assumption that patients discharged alive have no mortality due to covid-19 at a later date.

© 2020 Foy BM et al. *JAMA Network Open.*

Modified censoring protocol

When creating the Kaplan-Meier curves for the main study, patients who were discharged alive were assumed to have survived up until the final patient discharge (June 26th, 2020). This censoring protocol assumes that few high mortality-risk COVID-19+ in-patients were discharged, and that discharged patients who developed COVID-19 complications would have been readmitted. However, for completeness, in **eFigure 4** we present age and RDW-stratified Kaplan-Meier curves, censoring patients at the point of individual discharge. We note that this approach places a strong negative bias on the results, as healthier patients will likely have shorter hospital stays, and thus be censored earlier than the most at-risk patients. Despite this negative bias, elevated RDW is still associated with elevated mortality risk for all age groups. Note that this censoring does not affect **Table 2** in the manuscript, which already presents mortality rates at the point of discharge.

eFigure 4. Age and RDW-stratified mortality curves, censoring patients upon hospital discharge

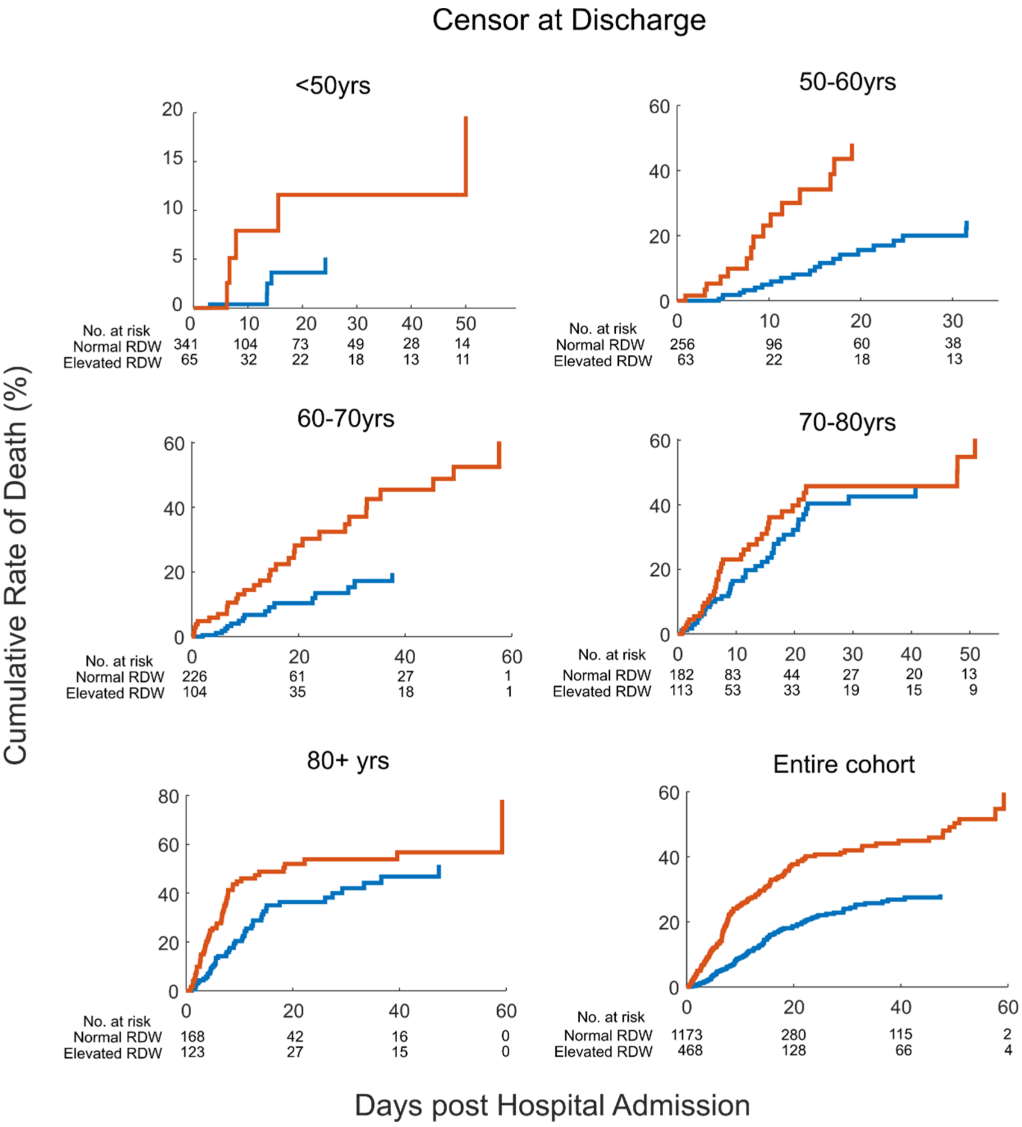

Note that at-risk numbers reflect cohort sizes remaining in the hospital at each given time point.

© 2020 Foy BM et al. *JAMA Network Open.*

Risk ratios for COVID-19+ stratified by age

Figure 2 presents Cox proportional-hazards risk ratios for RDW, accounting for age, race, ethnicity, absolute lymphocyte count, and D-dimer. However, as noted in **Table 2**, there appears to be an effect modification, whereby RDW elevation increases risk more significantly for younger patients than older patients. To investigate these potential effects in the results of **Figure 2**, in **eTable 3** we present proportional-hazards models for each separate age category. When considered as either a continuous variable or a discrete binary variable (RDW > 14.5%), RDW has a statistically significant risk ratio for all age groups except 70-80yrs, where RDW is significant in the continuous model but not the discrete model. No other variable had consistently significant multivariate risk ratios for multiple age groups. Similar to **Table 2**, an effect modification in the discrete model can be seen, with RDW having higher risk ratios for younger patient groups (<50yrs, 50-60yrs, 60-70yrs) than for older patient groups (70-80yrs, 80+yrs).

eTable 2. Multivariate Cox proportional-hazards analysis stratified by age

		Continuous			
		Multivariate		Univariate	
Age Group	Variable	Hazard Ratio	p-value	Hazard Ratio	p-value
<50yrs	RDW	1.13 (1.04-1.23)	0.003	1.13 (1.05-1.21)	0.001
	ALYMPH	1.06 (1.18-0.96)	0.25	1.01 (1.11-0.91)	0.92
	D-DIMER	1.00 (0.97-1.04)	0.81	1.01 (0.98-1.05)	0.51
	Race: Black/African American	2.26 (0.38-13.47)	0.37	1.63 (0.35-7.69)	0.53
	Ethnicity: Hispanic	1.98 (0.42-9.27)	0.39	0.90 (0.26-3.10)	0.87
50-60yrs	RDW	1.06 (1.02-1.11)	0.005	1.06 (1.02-1.11)	0.002
	ALYMPH	1.02 (1.09-0.96)	0.54	1.02 (1.09-0.96)	0.55
	D-DIMER	1.01 (0.99-1.03)	0.23	1.02 (1.00-1.04)	0.02
	Race: Black/African American	1.32 (0.55-3.15)	0.53	1.40 (0.67-2.90)	0.37
	Ethnicity: Hispanic	1.12 (0.50-2.50)	0.79	0.81 (0.40-1.64)	0.56
60-70yrs	RDW	1.19 (1.12-1.26)	<0.001	1.17 (1.11-1.24)	<0.001
	ALYMPH	1.02 (1.06-0.98)	0.31	1.00 (1.01-0.99)	0.77
	D-DIMER	1.00 (0.99-1.02)	0.63	1.01 (0.99-1.02)	0.25
	Race: Black/African American	1.12 (0.53-2.39)	0.76	1.56 (0.84-2.91)	0.16
	Ethnicity: Hispanic	1.38 (0.65-2.92)	0.40	0.84 (0.43-1.65)	0.62
70-80yrs	RDW	1.06 (1.01-1.12)	0.02	1.06 (1.01-1.12)	0.02
	ALYMPH	1.00 (1.02-0.99)	0.58	1.00 (1.02-0.99)	0.59
	D-DIMER	1.01 (0.99-1.02)	0.08	1.01 (1.00-1.02)	0.05
	Race: Black/AA	1.55 (0.86-2.80)	0.15	1.94 (1.17-3.23)	0.01
	Ethnicity: Hispanic	0.74 (0.37-1.46)	0.38	0.67 (0.35-1.26)	0.21
>= 80 yrs	RDW	1.11 (1.06-1.16)	<0.001	1.10 (1.05-1.15)	<0.001
	ALYMPH	1.01 (1.04-0.98)	0.46	1.01 (1.03-0.98)	0.49
	D-DIMER	1.00 (0.98-1.01)	0.62	1.00 (0.98-1.01)	0.55

© 2020 Foy BM et al. *JAMA Network Open.*

	Race: Black/African American	0.72 (0.37-1.38)	0.32	0.83 (0.45-1.51)	0.54
	Ethnicity: Hispanic	1.15 (0.60-2.19)	0.68	0.89 (0.48-1.67)	0.72
		Discrete			
		Multivariate		Univariate	
Age Group	Variable	Hazard Ratio	p-value	Hazard Ratio	p-value
<50yrs	RDW	5.50 (1.53-19.76)	0.009	5.51 (1.59-19.03)	0.007
	ALYMPH	1.18 (0.30-4.63)	0.82	1.30 (0.34-5.03)	0.70
	D-DIMER	0.89 (0.19-4.30)	0.89	1.21 (0.26-5.71)	0.81
	Race: Black/African American	1.35 (0.24-7.43)	0.73	1.63 (0.35-7.69)	0.53
	Ethnicity: Hispanic	1.20 (0.31-4.71)	0.79	0.90 (0.26-3.10)	0.87
50-60yrs	RDW	2.64 (1.31-5.30)	0.006	3.00 (1.53-5.86)	0.001
	ALYMPH	1.44 (0.74-2.82)	0.28	1.66 (0.86-3.21)	0.13
	D-DIMER	3.23 (1.66-6.31)	0.001	3.63 (1.89-6.98)	<0.001
	Race: Black/African American	0.97 (0.43-2.21)	0.95	1.40 (0.67-2.90)	0.37
	Ethnicity: Hispanic	1.02 (0.47-2.21)	0.97	0.81 (0.40-1.64)	0.56
60-70yrs	RDW	4.15 (2.28-7.55)	<0.001	3.96 (2.22-7.06)	<0.001
	ALYMPH	1.59 (0.89-2.84)	0.12	1.48 (0.84-2.61)	0.17
	D-DIMER	0.92 (0.48-1.76)	0.81	1.18 (0.62-2.23)	0.61
	Race: Black/African American	1.88 (0.96-3.70)	0.07	1.56 (0.84-2.91)	0.16
	Ethnicity: Hispanic	1.32 (0.64-2.73)	0.45	0.84 (0.43-1.65)	0.62
70-80yrs	RDW	1.41 (0.9-2.21)	0.13	1.45 (0.93-2.27)	0.11
	ALYMPH	0.99 (0.61-1.60)	0.95	0.96 (0.61-1.50)	0.85
	D-DIMER	1.13 (0.71-1.79)	0.62	1.19 (0.75-1.88)	0.46
	Race: Black/African American	1.82 (1.06-3.14)	0.03	1.94 (1.17-3.23)	0.01
	Ethnicity: Hispanic	0.76 (0.39-1.48)	0.42	0.67 (0.35-1.26)	0.21
>= 80 yrs	RDW	2.12 (1.43-3.15)	<0.001	1.92 (1.31-2.82)	<0.001
	ALYMPH	2.41 (1.61-3.60)	<0.001	2.30 (1.55-3.41)	<0.001
	D-DIMER	0.93 (0.63-1.38)	0.71	1.01 (0.68-1.49)	0.96
	Race: Black/African American	0.76 (0.41-1.41)	0.39	0.83 (0.45-1.51)	0.54
	Ethnicity: Hispanic	1.16 (0.61-2.19)	0.65	0.89 (0.48-1.67)	0.72

Legend: RDW – Red cell distribution width; ALYMPH – Absolute lymphocyte count. Race was coded such that risk ratios above 1 indicate elevated risk for those identifying as Black or African American, compared to all other identifications (White/Caucasian/Asian/Pacific Islander/Other/Unknown/Declined). Ethnicity was coded such that risk ratios above 1 indicate elevated risk for those identifying as Hispanic, compared to all other groups (Non-Hispanic/Unknown/Declined).

© 2020 Foy BM et al. *JAMA Network Open.*

Risk ratios of other common COVID-19+ comorbidities

Figure 2 of the main manuscript presents mortality risk ratios (both multivariate and univariate) for RDW in combination with age, race, ethnicity, absolute lymphocyte count (ALYMPH) and D-dimer. These covariates were chosen based on prior reports identifying them as risk factors for poor outcomes in COVID-19+ patients. Here we present risk ratios for these factors when modelled in conjunction with five important COVID-19 comorbidities: chronic obstructive pulmonary disease (COPD), diabetes mellitus (DM), hypertension (HTN), coronary artery disease (CAD), and chronic kidney disease (CKD). As in the main text, we consider RDW, ALYMPH, D-dimer, and age both as continuous variables (unit normalized in the same way as in the main text), and as discrete variables (with the same abnormality thresholds as in the main text). **eTable 4** shows risk ratios that are consistent with those in the main manuscript. Multivariate discrete risk ratios for RDW (>14.5%) are higher than the ratios of any of the comorbidities when using a 14.5% threshold. While the point estimate of the RDW risk ratio is lower in a continuous context than some of the comorbidities, it has a higher level of significance (lower p-value), and reflects the difference in comparison of a binary variable with a unit-normalized continuous variable (i.e. a different choice of unit normalization could increase the RDW risk ratio to be greater than those of the comorbidities). Four of the comorbidities (CAD, CKD, COPD, HTN) had significant risk ratios in a univariate context. However, only CKD and COPD retained significance in a multivariate model. These results support the conclusion that the association of RDW and mortality risk persists even when adjusting for common comorbidities.

eTable 3. Multivariate Cox proportional-hazards analysis including common comorbidities

	Multivariate continuous		Multivariate discrete		Univariate	
Variable	Risk Ratio	p-value	Risk Ratio	p-value	Risk Ratio	p-value
Age	1.59 (1.46-1.73)	< 0.001	3.07 (2.34-4.02)	< 0.001		
RDW	1.09 (1.07-1.12)	< 0.001	2.01 (1.57-2.57)	< 0.001		
ALYMPH	1.01 (1.02-0.99)	0.27	1.56 (1.22-1.99)	< 0.001		
D-DIMER	1.00 (1.00-1.01)	0.23	1.17 (0.91-1.51)	0.21		
Race: Black/African American	1.21 (0.87-1.69)	0.25	1.33 (0.98-1.81)	0.07		
Ethnicity: Hispanic	1.05 (0.75-1.48)	0.76	0.87 (0.63-1.22)	0.43		
CAD	1.01 (0.71-1.44)	0.94	1.10 (0.78-1.54)	0.60	1.93 (1.40-2.66)	< 0.001
CKD	1.68 (1.21-2.34)	0.002	1.66 (1.20-2.29)	0.002	2.65 (1.98-3.54)	< 0.001
COPD	1.88 (1.30-2.70)	0.001	1.69 (1.18-2.44)	0.005	2.81 (1.98-3.99)	< 0.001
DM	0.95 (0.68-1.32)	0.76	0.87 (0.63-1.20)	0.40	1.28 (0.96-1.70)	0.09
HTN	1.02 (0.77-1.35)	0.90	1.05 (0.80-1.4)	0.71	1.80 (1.41-2.30)	< 0.001

Legend: RDW – Red cell distribution width; ALYMPH – Absolute lymphocyte count; CAD – Coronary artery disease; CKD – chronic kidney disease; COPD – chronic obstructive pulmonary disorder; DM – Diabetes mellitus; HTN – hypertension. Race was coded such that risk ratios above 1 indicate elevated risk for those identifying as Black or African American, against all other identifications (White/Caucasian/Asian/Pacific Islander/Other/Unknown/Declined). Ethnicity was coded such that risk ratios above 1 indicate elevated risk for those identifying as Hispanic, comparative to all other groups (Non-Hispanic/Unknown/Declined).

© 2020 Foy BM et al. *JAMA Network Open.*

Risk ratios of other complete blood count measures

eTable 5 presents risk ratios using a multivariate proportional-hazards model for other complete blood count measures: hematocrit (HCT); hemoglobin (HGB); mean corpuscular hemoglobin (MCH); mean corpuscular hemoglobin concentration (MCHC); platelet count (PLT); and red blood cell count (RBC). For the continuous model, unit normalization was applied to each measure: HCT (1%), HGB (0.5g/dL), MCH (1pg), MCHC (0.5g/dL), PLT (10 * 10^3/µL), RBC (0.1 * 10^6/µL). For the discrete model, thresholds were applied using MGH reference intervals: HCT < 41(male)/36(female); HGB<13.5(m)/12(f); MCH<26; MCHC<31; PLT<150; RBC<4.5(m)/4.0(f). Consistent with the main manuscript, RDW was unit normalized (0.5%), and discretized with a threshold of RDW > 14.5%. In a univariate context, each blood count measure was significantly associated with mortality risk, except for MCH. However, in a multivariate model, PLT and RDW were the only blood count measures with significant risk ratios. In both the continuous and discrete case, RDW had a larger risk ratio than PLT. These results suggest that RDW elevation may be more significantly associated with mortality risk than changes in other blood count measures. Further investigation of the PLT count association is warranted. Note that WBC was excluded from this analysis due to the inclusion of absolute lymphocyte count.

eTable 4. Cox proportional-hazards analysis including blood count measures

	Continuous			
	Multivariate		Univariate	
Variable	Risk Ratio	p-value	Risk Ratio	p-value
Age	1.61 (1.47-1.77)	< 0.001	1.60 (1.49-1.72)	< 0.001
RDW	1.11 (1.08-1.14)	< 0.001	1.09 (1.07-1.10)	< 0.001
ALYMPH	1.01 (1.02-0.99)	0.252	1.01 (1.03-0.99)	0.23
D-DIMER	1.01 (1.00-1.01)	0.11	1.01 (1.01-1.02)	< 0.001
Race: Black/African American	1.19 (0.85-1.65)	0.31	1.30 (0.97-1.73)	0.08
Ethnicity: Hispanic	1.05 (0.75-1.48)	0.77	0.46 (0.34-0.63)	< 0.001
HCT	1.05 (0.71-1.56)	0.80	0.96 (0.94-0.98)	< 0.001
HGB	0.82 (0.47-1.44)	0.50	0.93 (0.90-0.95)	< 0.001
MCH	0.49 (0.19-1.22)	0.13	1.05 (1.00-1.10)	0.05
MCHC	1.42 (0.90-2.26)	0.13	0.87 (0.84-0.90)	< 0.001
PLT	0.97 (0.95-0.98)	< 0.001	0.96 (0.94-0.97)	< 0.001
RBC	1.09 (0.97-1.23)	0.16	0.96 (0.94-0.97)	< 0.001
	Discrete			
	Multivariate		Univariate	
Variable	Risk Ratio	p-value	Risk Ratio	p-value
Age	3.08 (2.36-4.02)	< 0.001	4.17 (3.25-5.35)	< 0.001
RDW	2.04 (1.55-2.69)	< 0.001	2.92 (2.30-3.69)	< 0.001
ALYMPH	1.49 (1.16-1.91)	0.002	1.91 (1.51-2.42)	< 0.001
D-DIMER	1.17 (0.92-1.51)	0.21	1.77 (1.39-2.26)	< 0.001
Race: Black/African American	1.38 (1.02-1.87)	0.04	1.30 (0.97-1.73)	0.08
Ethnicity: Hispanic	0.89 (0.64-1.24)	0.49	0.46 (0.34-0.63)	< 0.001

© 2020 Foy BM et al. *JAMA Network Open.*

HCT	0.85 (0.52-1.37)	0.50	1.63 (1.28-2.06)	< 0.001
HGB	1.09 (0.67-1.77)	0.72	1.78 (1.41-2.26)	< 0.001
MCH	0.70 (0.38-1.31)	0.27	1.00 (0.67-1.50)	1.00
MCHC	1.33 (0.94-1.88)	0.10	2.38 (1.75-3.24)	< 0.001
MCV	0.99 (0.50-1.93)	0.97	0.90 (0.57-1.41)	0.64
PLT	1.76 (1.37-2.25)	< 0.001	2.34 (1.84-2.98)	< 0.001
RBC	1.19 (0.79-1.79)	0.41	1.91 (1.50-2.42)	< 0.001

Legend: RDW – Red cell distribution width; ALYMPH – Absolute lymphocyte count; HCT – Hematocrit; HGB – Hemoglobin; MCH – Mean corpuscular hemoglobin; MCHC – Mean corpuscular hemoglobin concentration; PLT – platelet count; RBC – Red blood cell count.

© 2020 Foy BM et al. *JAMA Network Open.*

Mean corpuscular volume (MCV) changes throughout hospital stay

Figure 3 shows mean RDW trajectories for patients who had at least a one-week hospital stay (N=967), stratified by admission RDW and mortality. Since RDW is defined as the standard deviation of the RBC volume distribution divided by the MCV, a decrease in MCV is one explanation for an increase in RDW. To explore this possible explanation for the RDW changes, **eFigure 5** presents the corresponding MCV trajectories for the same patient groups. The MCV at admission in the elevated RDW group was lower by an average of 1.1 fL (87.9 v 89), and while this smaller MCV would be associated with an increased RDW, the effect should be small, raising RDW only by about 0.2%, compared to the ~3.0% actual difference in RDW between these groups in **Figure 3**. It is therefore likely that increased RBC volume variance is the major explanation for the elevated RDW in this cohort.

eFigure 5. Mean MCV trajectories for inpatients, stratified by RDW and mortality

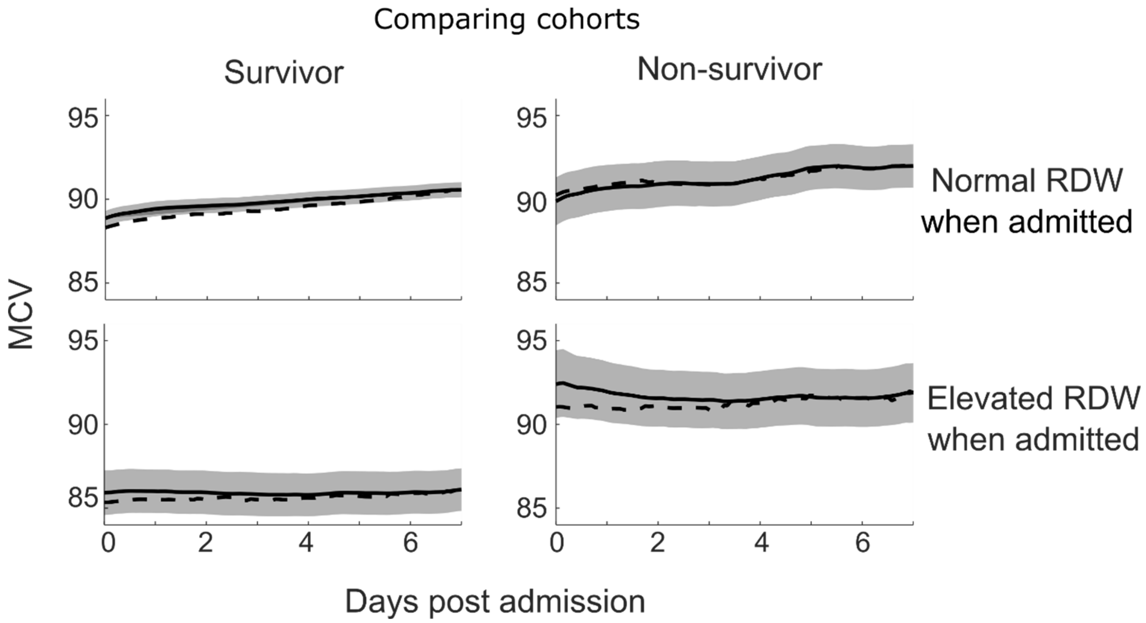

Mean trajectories (thick line) and 95% confidence intervals (colored region) are given for each patient group. For completeness, mean trajectories when including patients with shorter hospital stays (< 7 days) are also included (dashed line). The exclusion of shorter stay patients does not appear to alter the mean trajectories.

When calculating the mean trajectories, we excluded patients with a short hospital stay (< 7 days) to reduce the influence of patient discharges. In **eFigure 5** and **eFigure 6**, we compare these trajectories created without excluding short stay patients. To account for changes in cohort size (caused by patients being discharged prior to 1 week), the new trajectories were created by calculating the mean MCV and RDW values at each time point, based on the remaining patient cohort (i.e. those who had not yet been discharged). The comparisons in **eFigure 5-6** show that the exclusion of short-stay patients did not alter the patient trajectories in a way that would affect their interpretation. In particular, regardless of exclusion, patients who start with normal RDW but do not survive exhibit (on average) an RDW increase of ~1.5% over the first week of hospitalization.

© 2020 Foy BM et al. *JAMA Network Open.*

Note that all patients had at least two RDW measurements available, even those with short hospital stays. Of the 16 patients with a stay less than 24hrs, the 5 who survived had a mean RDW change of 0%, while the 11 who died experienced a mean RDW increase of 0.3%.

eFigure 6. Mean RDW trajectories for inpatients, stratified by RDW and mortality, including short stay patients

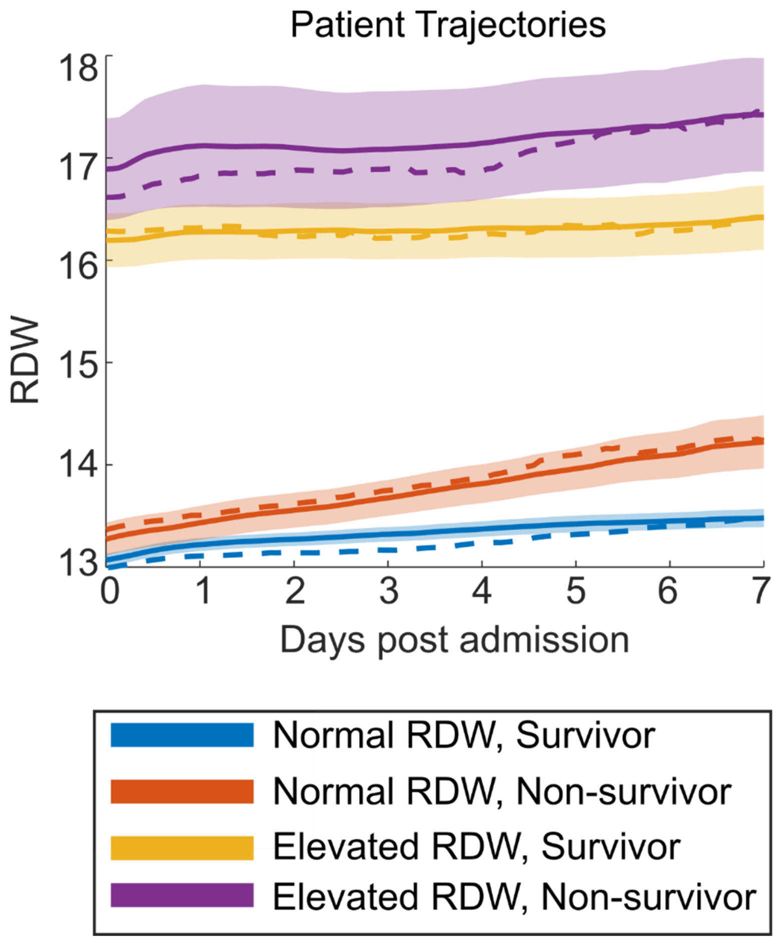

Mean trajectories (thick line) and 95% confidence intervals (colored region) are given for each patient group. For completeness, mean trajectories when including patients with shorter hospital stays (< 7 days) are also included (dashed line). The exclusion of shorter stay patients does not appear to alter trajectories meaningfully.

© 2020 Foy BM et al. *JAMA Network Open.*
